# Stool- and Blood-Associated Colorectal Cancer Biomarkers: A Systematic Review

**DOI:** 10.3390/cancers18010096

**Published:** 2025-12-27

**Authors:** Pumelela Hallom, Pragalathan Naidoo, Sibusiso Senzani, Sayed S. Kader, Zilungile L. Mkhize-Kwitshana

**Affiliations:** 1 Department of Medical Microbiology, School of Medicine, College of Health Sciences, University of KwaZulu-Natal, Nelson R. Mandela Medical School Campus, Durban 4001, South Africa; naidoop5@ukzn.ac.za (P.N.); senzanis@ukzn.ac.za (S.S.); 2 Division of Research Capacity Development (RCD), South African Medical Research Council (SAMRC), Tygerberg, Cape Town 7505, South Africa; 3 Department of Surgery, University of KwaZulu Natal, Congella, Durban 4013, South Africa; kaders@ukzn.ac.za; 4 Biomedical Sciences Department, School of Life and Consumer Sciences, College of Agriculture and Environmental Sciences, University of South Africa, Florida Campus, Johannesburg 1710, South Africa

**Keywords:** blood, stool, biomarkers, colorectal cancer, non-invasive screening methods

## Abstract

**Background/Objectives**: Colorectal cancer (CRC) is a major contributor to cancer-related deaths worldwide. While existing screening tools are effective, their high cost and limited availability restrict widespread adoption, particularly in low- and middle-income settings. The identification of affordable, non-invasive biomarkers is therefore critical to improve early CRC detection and survival outcomes. **Methods**: A systematic literature search was performed through PubMed, ScienceDirect, Medline, ISI Web of Knowledge, and Google Scholar to identify studies reporting stool- and blood-based biomarkers for CRC detection. Data were extracted using a standardized template, including study details, specimen type, detection method, and diagnostic performance parameters such as sensitivity and specificity. **Results**: DNA methylation biomarkers demonstrated high diagnostic potential. *Syndecan 2 (SDC2)* and *Short Stature Homeobox 2 (SHOX2)* achieved a combined stool sensitivity of 91.35%. Other methylation markers, including *NDRG4*, *SEPT9*, and *BCAT1*, showed a composite sensitivity of 82.7%. Plasma-based methylation markers such as *GATA5*, *FOXE1*, and *SYNE1* reported sensitivities ranging from 18–47% and specificities of 93–99%. Hypermethylation of *SFRP2* and *WIF-1* achieved 81.3% sensitivity in CRC and precursor lesions. *Matrix metalloproteinases* (*MMP-2* and *MMP-9*) were elevated in CRC patients, with stool *MMP-9* yielding 72.2% sensitivity and 95% specificity. A stool gene panel (*UBE2N*, *IMPDH1*, *DYNC1LI1*, *HRASLS2*) reached 96.6% sensitivity and 89.7% specificity, while a methylation-based panel (*ALX4*, *BMP3*, *NPTX2*, *RARB*, *SDC2*, *SEPT9*, *VIM*) achieved 90.7% sensitivity. MicroRNAs (*miR-21*, *miR-92a*, *miR-223*, *miR-182*) showed excellent diagnostic performance, with sensitivities exceeding 96% and specificities above 75%. **Conclusions**: DNA methylation and microRNA biomarkers hold strong promises for non-invasive CRC screening. Multi-marker panels demonstrate superior diagnostic accuracy and may provide a cost-effective, scalable approach for early CRC detection in resource-limited settings.

## Simple Summary

Colorectal cancer is one of the most common and deadly cancers worldwide, but many people are diagnosed too late because current screening tests are expensive, invasive, or not easily available. Early detection greatly increases the chances of successful treatment. This research aims to find simple, accurate, and non-invasive ways to detect colorectal cancer using biological markers found in blood and stool. These markers can reveal early changes in the body that happen before cancer develops. By reviewing the most promising blood and stool markers, this study highlights potential tools that could make screening easier and more accessible for everyone. The findings may guide future research toward developing affordable tests that can detect colorectal cancer earlier, reduce deaths, and improve global health outcomes.

## 1. Introduction

Colorectal cancer is a significant global health burden, ranking as the third most diagnosed cancer and the second leading cause of cancer mortality [1]. It accounts for approximately 10.2% of new cancer cases and 9.2% of cancer-related deaths annually [1]. Early-stage CRC is frequently asymptomatic, leading to diagnosis at advanced stages when treatment efficacy declines. The 5-year survival rate exceeds 90% for stage I CRC but drops to 11–15% for stage IV disease [2,3], underscoring the critical importance of early detection and timely intervention.

Current CRC detection relies primarily on endoscopic techniques (colonoscopy, sigmoidoscopy), imaging modalities such as computed tomography (CT) colonography, and non-invasive fecal tests, including the guaiac-based fecal occult blood test (gFOBT) and fecal immunochemical test (FIT) [4,5,6]. While colonoscopy is highly sensitive and allows for both diagnosis and removal of precancerous lesions, it is invasive, costly, and requires bowel preparation, which may reduce patient compliance [5,6]. CT colonography provides a non-invasive imaging alternative, yet its availability is limited in many healthcare settings. Fecal-based tests like FIT are convenient and non-invasive but exhibit limited sensitivity, particularly for detecting precancerous lesions (27–29%) and right-sided tumors (65.8–75%) [7,8,9,10,11,12]. Collectively, these limitations highlight the need for more accurate, accessible, and patient-friendly diagnostic approaches.

To address these challenges, attention has increasingly turned to molecular biomarkers detectable in stool or blood, which reflect the genetic and epigenetic alterations that occur during CRC development [13,14,15,16,17,18,19,20,21,22,23,24,25]. These biomarkers include DNA methylation markers, microRNAs (miRNAs), tumor suppressor genes, and inflammatory proteins [16,17,18,19,20,21,22,23]. DNA methylation, an epigenetic modification associated with early tumorigenesis, can be detected in exfoliated colonocytes in stool, providing a potential non-invasive avenue for early CRC detection. Stool-based DNA methylation markers such as *Tissue Factor Pathway Inhibitor 2* (*TFPI2*), *VIM*, *SFRP2*, *NDRG4*, *BMP3*, and *SDC2* have shown promising diagnostic performance in several studies [19,20,21,22]. Circulating miRNAs, including *miR-21* and *miR-223*, are stable in blood and represent another non-invasive biomarker class, capable of reflecting tumor biology and disease progression [22,23]. The identification of effective biomarkers for early CRC detection or monitoring is important because it could enable the detection of precancerous adenomas, which are fully amenable to endoscopic removal, a critical step in preventing the development of invasive and metastatic colorectal cancer [24,25]. This systematic review aims to identify, summarize, and evaluate stool- and blood-based biomarkers associated with CRC, focusing on those with the highest potential for non-invasive early detection.

## 2. Materials and Methods

A systematic literature search was conducted to collect data regarding biomarkers in stool and blood samples that might be promising for early detection of CRC. A narrative approach was followed to review relevant and available data on this topic. This systematic review has been registered under International Prospective Register of Systematic Review (PROSPERO) with reference ID: CRD42024572827.

### 2.1. Literature Search Strategy

The Preferred Reporting Items for Systematic Reviews and Meta-Analyses (PRISMA) guidelines were used to analyze and report relevant data in this systematic review. A systematic literature search was performed in PubMed, ScienceDirect, Medline, Institute for Scientific Information (ISI) Web of Knowledge, and Google Scholar databases using the following search keywords: ‘Stool/fecal/faecal biomarkers’, ‘Blood biomarkers’, colorectal cancer/CRC/colon cancer/rectal cancer/intestinal cancer’, ‘Stool biomarker and colorectal cancer or CRC’, ‘Blood biomarkers and colorectal cancer or CRC’. All globally published studies in the English language between January 2000 and September 2025 were included in this study.

### 2.2. Study Selection, Quality of Studies, and Data Extraction

The selection of appropriate literature for this study followed a structured process. Initially, titles, abstracts, and full texts were reviewed based on predefined inclusion and exclusion criteria by the primary author (P.H.). The eligibility of the selected literature was subsequently verified and cross-checked for discrepancies and duplicates by three co-authors (P.N., S.S., and Z.L.M.K.). The quality of the extracted data was categorized into four levels: high, moderate, low, and very low quality, as evaluated using the Grading of Recommendations, Assessment, Development, and Evaluations (GRADE) approach [26,27].

Data were extracted from the eligible studies in a pre-defined form, including the first author’s name and publication year, sample population, specimen type, detection method, and diagnostic performance characteristics (sensitivity (%) and specificity (%) of biomarkers in CRC. The studies were qualified for eligibility according to pre-specified inclusion criteria.

**Figure 1:**
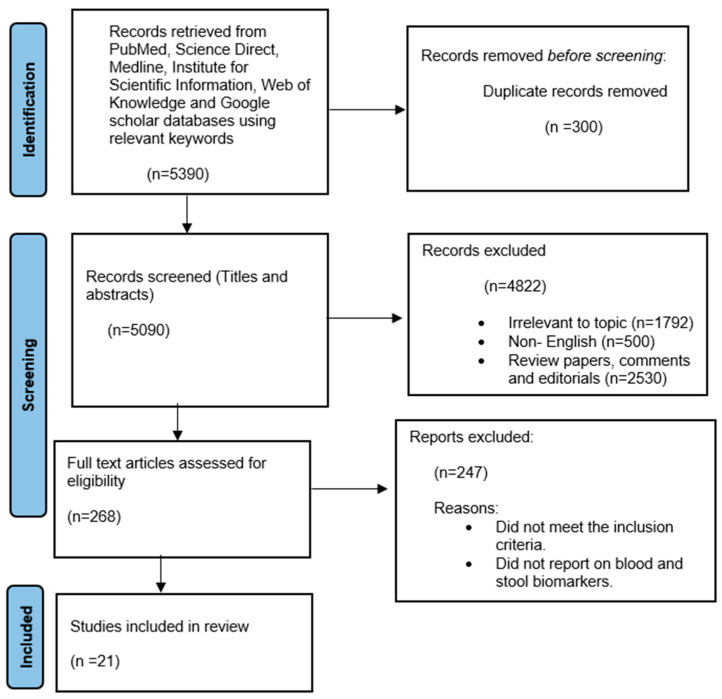
Preferred Reporting Items for Systematic Reviews and Meta-Analyses (PRISMA) flow diagram highlighting the article selection process for writing up the systematic review.

Upon searching the above-mentioned databases with the relevant search criteria, a total of 5390 articles were found. Further evaluation revealed a total of 300 articles were duplicates, leaving a total of 5090 articles to be further assessed, considering the qualifying requirements. Articles that were not relevant were excluded (4822), and 268 full articles were assessed for eligibility. Ultimately, 21 of the 268 articles were selected based on stool- and blood-associated CRC biomarkers. Figure 1 shows a systematic flow diagram for selecting the articles selected for this systematic review according to initial identification, screening, eligibility, and final selection.

### 2.3. Inclusion Criteria

- Literature reporting on stool and blood biomarkers for CRC.
- Literature published in English in all countries from January 2000 to September 2025.
- Cross-sectional studies, case–control studies, and cohort-appropriate studies.
- Literature published in the five databases mentioned above.

### 2.4. Exclusion Criteria

- Literature published before January 2000.
- Articles not published in English.
- Comments, editorials, and Reviews. Articles not relevant to stool and blood biomarkers for diagnosis of CRC.

## 3. Results

An overview of the studies included in the systematic review is highlighted in Table 1. Detailed information on the specific biomarkers analyzed, sample sources, and molecular techniques employed for CRC detection is summarized in Table 2. CRC. Table 3 summarizes the optimal stool and serum biomarkers identified in the review, along with their reported sensitivity and specificity.

## 4. Discussion

This systematic review evaluated 21 publications examining stool- and blood-based biomarkers for colorectal cancer, highlighting their potential for early detection and non-invasive screening. The findings underscore the promise of DNA methylation markers, microRNAs, protein biomarkers, and microbial signatures, particularly when used in combination, to improve diagnostic accuracy.

### 4.1. DNA Methylation Biomarkers

DNA methylation remains one of the most studied approaches for non-invasive CRC detection. Markers such as *Syndecan-2* (*SDC2*), *Short Stature Homeobox 2* (*SHOX2*), *N-Myc downstream-regulated gene 4* (*NDRG4*), *Septin9* (*SEPT9*), and *Branched-Chain Amino Acid Transaminase 1* (*BCAT1*) showed high sensitivity and specificity, especially in stool samples. For instance, *SDC2* and *SHOX2* methylation in stool achieved sensitivity of 91.35% and 89.5%, respectively, and 93.35% when combined, with specificities exceeding 85% [28]. Similarly, *NDRG4* methylation in stool showed promising sensitivity (76.2%) and specificity (89.1%), though its performance in blood was lower, highlighting the superiority of stool-based detection for some methylation biomarkers [32,42]. Several studies demonstrated that methylation patterns of *SEPT9*, *SDC2*, and *BCAT1* in plasma (circulating tumor DNA) could distinguish CRC from benign polyps with high diagnostic accuracy (AUC = 0.914) [33]. Multi-gene panels further improved performance, emphasizing the value of combining targets to capture tumor heterogeneity and enhance early detection [31,48]. These findings suggest that DNA methylation biomarkers, particularly when assessed in stool or as part of composite panels, can reliably detect early-stage CRC and even precancerous lesions [28,29,34].

### 4.2. MicroRNAs (miRNAs)

miRNAs have emerged as powerful biomarkers due to their stability in biofluids and their role in tumor biology. *miR-21*, *miR-92a*, *miR-223*, and *miR-182* were consistently upregulated in CRC patients in both stool and plasma samples. Individually, these miRNAs showed good sensitivity and specificity, but combination analysis (e.g., *miR-223* + *miR-92a*) yielded the highest diagnostic accuracy (sensitivity 96.8%, specificity 75%, AUC 0.907) [31,35,40]. The oncogenic role of miRNAs, particularly *miR-223* and *miR-182*, also provides mechanistic insight; their upregulation promotes cell proliferation, invasion, and survival, linking biomarker detection to tumor biology [48,49,50]. These data suggest that miRNAs can serve as complementary diagnostic tools alongside DNA methylation, enhancing non-invasive CRC detection and potentially informing prognosis [30,39,46].

### 4.3. Protein Biomarkers

Protein-based biomarkers, including matrix metalloproteinases (MMP-2, MMP-9), Insulin-Like Growth Factor Binding Protein 2 (IGFBP2), Dickkopf-related protein 3 (DKK3), and Pyruvate Kinase M2 (PKM2), showed variable but clinically relevant performance. Fecal MMP-9 demonstrated sensitivity ranging from 72.2% to 89.3% and specificities from 91.2% to 95%, although some studies reported inconsistent results [51,52,53]. Serum levels of IGFBP2 and PKM2 were elevated in CRC patients, with a combined sensitivity and specificity of 73% and 95%, respectively [54]. These protein biomarkers reflect tumor-associated inflammation, extracellular matrix remodeling, and metabolic dysregulation, providing functional insights that complement genetic and epigenetic markers [21,55,56]. However, variability across studies highlights the need for standardized assays and validation in larger cohorts [52,53].

### 4.4. Microbial Biomarkers

The gut microbiome, particularly *Fusobacterium nucleatum* (*F. nucleatum*), has emerged as a promising CRC biomarker. Significantly higher levels of *F. nucleatum* were observed in CRC tissues and stool, and combining microbial detection with clinical parameters (e.g., fecal occult blood, CEA, CA19-9) improved diagnostic performance (AUC = 0.87) [38,45]. These findings suggest that microbial biomarkers can enhance non-invasive CRC detection when integrated into multi-parameter screening strategies.

### 4.5. Multi-Marker and Combination Panels

Several studies emphasized the superior performance of multi-marker panels over single biomarkers. For example, combining DNA methylation markers such as *Secreted frizzled-related protein 2* and *Wnt inhibitory factor 1* (*SFRP2* + *WIF-1*), miRNAs (*miR-21* + *miR-29a* + *miR-92a*), or ctDNA methylation with protein markers and fecal tests improved both sensitivity and specificity [31,39,41,48]. This approach addresses the inherent heterogeneity of CRC and increases the likelihood of detecting early-stage and precancerous lesions.

### 4.6. Limitations and Future Directions

A notable limitation of the current evidence is that many studies were conducted on small or ethnically homogeneous cohorts, which may restrict the generalizability of the findings. The diagnostic performance of several stool- and blood-based biomarkers has not been validated across diverse populations, and differences in genetic background, environmental exposures, and lifestyle factors may influence biomarker expression and methylation patterns.

Many studies were also limited by small sample sizes, single-center designs, and methodological variability (e.g., assay type, sample handling, cut-offs), which introduce potential bias and may affect reported sensitivity and specificity. Potential publication bias could further overestimate the diagnostic performance of some biomarkers. In addition, there is significant methodological heterogeneity among studies, including differences in DNA extraction kits, PCR protocols, methylation detection assays, and sample processing methods, which complicate comparisons and reduce reproducibility, representing a significant barrier to clinical translation.

While high specificity (>90%) was frequently reported, most studies employed case–control designs with healthy participants as controls. This may overestimate real-world specificity, as it does not fully account for common gastrointestinal conditions such as inflammatory bowel disease, diverticulitis, or benign polyps, which are important for clinical decision-making. Future studies should therefore include disease control groups with common benign gastrointestinal pathologies to provide a more realistic assessment of diagnostic specificity in a true screening population.

A further important limitation, which precluded quantitative meta-analysis, is the extensive heterogeneity observed across the studies included. This heterogeneity is clinical (diverse populations, disease stages), methodological (varying assay protocols, sample processing techniques, and positivity thresholds), and statistical (inconsistent reporting of essential data for pooling). The biomarker field for CRC is also characterized by rapid evolution, with many studies investigating novel, distinct targets or unique multi-marker panels. Consequently, there were insufficient homogeneous studies for any single biomarker to perform a statistically robust and clinically meaningful meta-analysis. Attempting to pool data under these circumstances would violate the core assumptions of meta-analytic models and generate unreliable summary estimates. Therefore, this review provides a comprehensive qualitative and comparative synthesis. We emphasize that the reported sensitivities and specificities must be interpreted within the specific context of each primary study.

Taken together, these factors suggest that while some biomarkers show promising results, the overall certainty of the evidence is limited, and conclusions should be interpreted with caution. There is an urgent need for standardized, universally accepted protocols, as well as large, multi-ethnic, population-based validation studies, to ensure the reliability, reproducibility, and clinical applicability of stool- and blood-based CRC biomarkers. Future research should prioritize standardized protocols and comprehensive data reporting to facilitate definitive quantitative synthesis.

## 5. Conclusions

This systematic review highlights the potential of stool- and blood-based biomarkers, including DNA methylation markers, microRNAs, protein biomarkers, and microbial signatures, for non-invasive early detection of colorectal cancer. While several biomarkers demonstrated high sensitivity and specificity, the overall certainty of evidence is limited due to small, ethnically homogeneous cohorts; methodological heterogeneity; and the reliance on healthy controls, which may overestimate real-world diagnostic performance. The findings underscore the critical need for standardized protocols, multi-marker panels, and validation in large, multi-ethnic, population-based studies, including participants with other gastrointestinal conditions, to confirm clinical utility. These efforts are essential to translate promising biomarkers into reliable, broadly applicable screening tools for early CRC detection.

## Figures and Tables

**Table 1:** An overview of all articles included in this systematic review investigating the efficacy of blood and stool biomarkers for non-invasive CRC screening.

Author (Subgroup)		Title	Study Population	Sample Type
DNA Methylation				
	[28]	“Novel DNA methylation biomarkers in stool and blood for early detection of colorectal cancer and precancerous lesions”	Samples were collected from hospitalized patients in the Department of Gastrointestinal Surgery of Renji Hospital, Shanghai Jiaotong University School of Medicine. Of 515 participants, 422 were analyzed; exclusions included incomplete data (12), collection issues (25), and insufficient genes (32). An additional 24 patients with interfering diseases had DNA methylation tests.	Stool Blood Tissue
	[29]	“Feasibility of quantifying *SDC2* methylation in stool DNA for early detection of colorectal cancer”	Stool samples were collected from participants at the Cancer Center of Yonsei University College of Medicine and Dongguk University Ilsan Hospital in South Korea. CRC confirmed (50), adenomatous polyps (21) and healthy patients (22).	Stool
	[30]	“Highly sensitive fecal DNA testing of *NDRG4* 12b methylation is a promising marker for detection of colorectal precancerosis”	Patients enrolled at the First Affiliated Hospital of Nanjing Medical University, a total of 238 individuals; 199 had fully evaluable results.	Stool
	[31]	“Detection of promoter hypermethylation of Wnt antagonist genes in fecal samples for diagnosis of early colorectal cancer”	Samples collected from patients with sporadic CRC, benign colorectal diseases, and 30 endoscopically normal patients undergoing surgery and endoscopy at the Zhongnan Hospital in Wuhan.	Stool
	[32]	“Quantitative detection of methylated *NDRG4* gene as a candidate biomarker for diagnosis of colorectal cancer”	A total of 87 CRC patients were recruited from the General Hospital of PLA in Beijing, China. A control group of 16 age-matched healthy subjects was included. Colon cancer (56) and rectal cancer (31)	Stool Blood Urine Tissue
	[33]	“Detection of Circulating Tumor DNA Methylation in Diagnosis of Colorectal Cancer”	Patients diagnosed with CRC and colorectal polyps through colonoscopy at Shanghai East Hospital from March to August 2019. CRC confirmed (104), colorectal polyps (130) and healthy patients (130).	Blood
	[34]	“Genome-Wide Identification and Validation of a Novel Methylation Biomarker, *SDC2*, for Blood-Based Detection of Colorectal Cancer”	Samples collected from CRC patients during surgery (all stages) at Yonsei University College of Medicine Cancer Center. Normal controls from healthy participants and European stage I–II CRC patients.	Blood Tissue
miRNA				
	[35]	“*MicroRNA-223* and *microRNA-92a* in stool and plasma samples act as complementary biomarkers to increase colorectal cancer detection”	291 CRC patients diagnosed at Chang Gung Memorial Hospital, Taiwan; 62 patients in training group, 229 in test group; 452 healthy controls recruited.	Stool Plasma
	[36]	“Investigation of *MicroRNA-21* Expression Levels in Serum and Stool as a Potential Non-Invasive Biomarker for Diagnosis of Colorectal Cancer”	40 CRC patients and 40 healthy controls, blood and stool samples from Shariati Hospital, Tehran, Iran.	Stool Serum
	[37]	“Fecal microRNAs as novel biomarkers for colon cancer screening”	Samples included 10 individuals with normal colonoscopy, 9 patients with adenomas, 10 CRC patients; selected from 303 fecal samples collected at Okayama University Hospital, Japan.	Stool
	[38]	“Role of *MicroRNA-223* and *microRNA-182* as Novel Biomarkers in Early Detection of Colorectal Cancer”	Case–control study at Aswan University Hospital; 65 participants (35 CRC cases, 30 controls)	Serum
	[39]	“Serum micro-RNA Identifies Early-Stage Colorectal Cancer in a Multi-Ethnic Population”	Sera collected from 18 healthy controls and 73 CRC cases at 4 sites in Hawaii and Japan.	Serum
	[40]	“Machine-learning-based Analysis Identifies miRNA Expression Profile for Diagnosis and Prediction of Colorectal Cancer: A Preliminary Study”	Serum samples collected from 8 CRC patients and 10 age- and sex-matched control patients	Serum
**Proteins**				
	[41]	“Blood-Based Protein Biomarker Panel for the Detection of Colorectal Cancer”	Patients with newly diagnosed CRC recruited from Victorian Cancer Biobank, Australia (2005–2011).	Blood
	[42]	“Faecal Diagnostic Biomarkers for Colorectal Cancer”	216 patients from Complexo Hospitalario Universitario de Ourense underwent colonoscopy and categorized into 4 groups.	Stool
	[43]	“The Use of M2-Pyruvate Kinase as a Stool Biomarker for Detection of Colorectal Cancer in Tertiary Teaching Hospital: A Comparative Study”	Prospective study at Hospital Universiti Sains Malaysia (HUSM) from September 2014 to January 2016; patients undergoing colonoscopy.	Stool
Gene Panels/Multi-gene Assays				
	[44]	“Potential prognostic and predictive value of *UBE2N*, *IMPDH1*, *DYNC1LI1* and *HRASLS2* in colorectal cancer stool specimens”	Stool samples from 58 CRC patients and 29 healthy individuals at Sijhih Cathay General Hospital	Stool
	[45]	“Improved diagnosis of colorectal cancer using combined biomarkers including *Fusobacterium nucleatum*, fecal occult blood, transferrin, CEA, CA19-9, gender, and age”	Samples were collected from 130 patients from Shanghai General Hospital (59 CRC patients and 71 healthy controls).	-Stool -Serum
	[46]	“Hypermethylated DNA, a circulating biomarker for colorectal cancer detection”	Patients at Aalborg University Hospital: 193 CRC patients and 102 controls	Blood
	[47]	“Spectrin Repeat Containing Nuclear Envelope 1 and Forkhead Box Protein E1 Are Promising Markers for the Detection of Colorectal Cancer in Blood”	220 plasma samples from CRC patients across multiple centers in Germany; 664 controls	Blood, Plasma

CRC: colorectal cancer; NDRG4: N-Myc Downstream-Regulated Gene 4; SDC2: Syndecan-2; UBE2N: Ubiquitin-Conjugating Enzyme E2 N; IMPDH1: Inosine Monophosphate Dehydrogenase 1; DYNC1LI1: Dynein Cytoplasmic 1 Light Intermediate Chain 1; HRASLS2: HRAS Like Suppressor 2.

**Table 2:** A comprehensive summary of articles included, identifying stool and blood biomarkers that can be used for early detection of colorectal cancer.

Author		Aim of Study	Sample Type	Molecular Methods	Major Findings	Clinical Stage
DNA Methylation						
	[28]	To explore novel and valuable DNA methylation biomarkers for CRC and precancerous lesions	Stool Blood Tissue	- DNA isolation from stools, blood, and tissue samples - Bisulfite conversion to transform the extracted DNA samples with sulfite. - Quantitative Methylation-specific PCR (qMSP) performed using DNA from sulfite-transformed samples. - The detection limits of the methylation biomarker assay were established using plasmids containing sequences of candidate CpG sites, which Nanjing Kingsray Biotechnology Co. (Nanjing, China) constructed	In stool samples, cg13096260 and cg12993163 showed high diagnostic performance for CRC. Overall sensitivity was 91.35% for cg13096260 and 89.5% for cg12993163, with specificities of 93.33% and 85.83%. For early-stage CRC, sensitivities reached 93.62% for cg13096260 and 92.55% for cg12993163, while for advanced adenomas, they were 63.04% and 82.6%. The combined diagnostic model achieved 93.83% overall sensitivity, 96.81% for early-stage CRC, 71.74% for advanced adenomas, and 92.5% specificity. In blood, both markers showed moderate performance around 70–80%. Stool detection of these markers is a promising strategy for early CRC and precancerous lesion screening.	Early-stage CRC: 93–96%; Advanced adenomas: 63–82%
	[29]	To investigate the feasibility of quantifying SDC2 methylation in stool DNA for the early detection of CRC	Stool	- DNA isolation from tissues, cell lines, and stool. - Bisulfite treatment - Bisulfite-pyrosequencing methylation assay of SDC2 gene in tissues - Analytic performance of meSDC2 LTE-qMSP - Methylation measurement in stool DNA by meSDC2 LTE-qMSP	A stool DNA test for SDC2 methylation showed increasing positivity with lesion severity: 0% in normal tissue, 90.6% in adenomatous polyps, 94.1% in hyperplastic polyps, and 100% in primary CRC tumors. Methylation levels rose significantly with lesion progression (*p* < 0.01). The test achieved 90.0% sensitivity for CRC, 33.3% for small polyps, and 90.9% specificity, based on LTE-qMSP analysis of 50 CRC patients, 21 precancerous lesions, and 22 healthy controls.	CRC stages I–IV
	[33]	To evaluate the effectiveness of a novel DNA methylation panel comprising *Septin9*, *SDC2*, and *BCAT1* for the detection of CRC and colorectal polyps in patient plasma samples.	Blood	- DNA isolation from plasma - Bisulfite conversion - RT-PCR	CRC patients showed elevated ctDNA methylation of three genes compared with polyps and healthy controls. The composite methylation score correlated with tumor stage but not location. BCAT1 and Septin9 distinguished CRC from polyps with 83.7% sensitivity and 93.9% specificity. Combining the methylation score with CEA and fecal hemoglobin testing further improved diagnostic accuracy.	Early- & late-stage
	[32]	To analyze the sensitivity and specificity of methylated NDRG4 gene expression for use as a biomarker in CRC	Tissue Blood, Urine Stool	- DNA isolation from stool, total blood and tissue - Bisulfite modification of genomic DNA - n-MSP to identify the expression of NDRG4 genes from blood, urine, stool and tissue. - Polyacrylamide gel electrophoresis,	NDRG4 methylation showed higher diagnostic accuracy in fecal (76.2% sensitivity, 89.1% specificity) and urine samples (72.6% sensitivity, 85% specificity) than in blood (54.8% sensitivity, 78.1% specificity). Detecting NDRG4 methylation in feces and urine is more effective for CRC diagnosis, and combining both sample types may further improve detection.	Stage not fully detailed
	[31]	To investigate the feasibility of detecting aberrantly hypermethylated Wnt-antagonist gene promoters (SFRP2 and WIF-1) in fecal DNA as non-invasive biomarkers for early CRC	Stool	- DNA extraction from stool samples - Bisulfite modification - Methylation-specific PCR analysis	Fecal hypermethylation of SFRP2 and WIF-1 was detected in CRC (56.3% and 60.4%) and adenoma patients (51.4% and 45.7%). Screening for both genes together improved detection, achieving sensitivity of 81.3% for CRC, 80.0% for advanced adenomas, and 25.0% for hyperplastic polyps, with 96.7% specificity for distinguishing CRC from benign colorectal conditions.	Early adenomas: 80%
	[30]	To research and validate techniques for extracting DNA from human genomes, assess the sensitivity and specificity of known nucleic acid markers associated with intestinal malignancy in Chinese patients with early colorectal cancer and identify adenoma-specific biomarkers in human DNA extracted from fecal samples.	Stool	- DNA isolation from stool samples - Bisulfite treatment - qMSP - Fecal occult blood testing on the same stool sample used for the DNA test	NDRG4 12b methylation detected advanced adenomatous polyps with 85.7% sensitivity and 70.8% specificity, and non-advanced polyps with 62.6% sensitivity. Compared to FOBT, it showed significantly better detection of advanced polyps (85.7% vs. 42.9%). The ROC curve for adenoma detection had an AUC of 0.807, indicating good diagnostic performance.	Early adenomas
	[34]	To investigate a specific subset of genes aberrantly methylated in primary tumor of CRC	Tissue Blood	- DNA isolation from serum, tissue specimens and cell lines. - CpG DNA Microarray Analysis in combination with the enrichment of methylated DNA through Media utilizing a recombinant protein domain (MBD2bt). - Bisulfite Treatment and DNA Purification. - Quantitative Bisulfite Pyrosequencing Analysis to quantify methylation levels of target genes in cell lines - and tissues. - qMSP	SDC2 methylation was identified as a potential biomarker for early CRC detection. In 139 CRC tissue samples, 97.8% showed aberrant SDC2 methylation. In serum, quantitative methylation-specific PCR detected CRC with 87.0% sensitivity and 95.2% specificity, including 92.3% sensitivity for stage I, supporting its use as a blood-based test for early CRC detection.	Stages I–IV
MicroRNA						
	[35]	To establish a 46-miRNA multiplex RT-qPCR method, and efficiently examine two clinically accessible samples: stool from a fecal occult blood test and EDTA plasma.	Stool Plasma	- RNA extraction from plasma and stool samples. - Stem-loop reverse transcription-polymerase chain reaction (RT-PCR) to quantify miRNA. - Quantitative polymerase chain reaction to quantify individual miRNAs.	A study of 62 tissue, 447 stool, and 398 plasma samples from CRC patients and healthy controls found strong correlations in miRNA expression across paired tumor, stool, and plasma specimens. Five stool miRNAs and eleven plasma miRNAs showed good discriminatory ability (AUC > 0.7) and were validated in an independent cohort. The combination of miR-223 and miR-92a, detectable in both stool and plasma, provided the best diagnostic performance, achieving 96.8% sensitivity, 75% specificity, and an AUC of 0.907. This resulted in a two-miRNA biosignature with high sensitivity for CRC detection.	Early- & late-stage CRC
	[38]	To determine the role of miR-223 and miR-182 as novel biomarkers for early detection and prognosis of CRC	Serum	- Total RNA extraction from sera - c-DNA Synthesis - qRT-PCR	Significant differences in biomarker levels were observed between CRC patients and controls, with ROC analysis showing strong diagnostic performance. miR-223 achieved 97.1% sensitivity and 96.7% specificity, while miR-182 showed 98% sensitivity and 96% specificity. Both miR-223 and miR-182 were identified as reliable biomarkers for early CRC diagnosis and prognosis, with higher expression levels associated with increased risk of disease progression and earlier detectability.	Early-stage detection feasible
	[36]	To evaluate the miR-21 expression level using real-time quantitative RT-PCR (qRT-PCR), to elucidate the clinical significance and potential efficiency of miR-21 as a valuable biomarker	Stool Serum	- RNA extraction from serum and stool samples - cDNA synthesis	miR-21 was upregulated in both blood and stool of CRC patients. Using qRT-PCR, blood miR-21 showed 86.1% sensitivity and 73.0% specificity for CRC detection. Stool miR-21 achieved 88.1% sensitivity and 81.6% specificity and could effectively distinguish advanced CRC (stages III–IV) from early-stage disease (stages I–II), highlighting its potential as a non-invasive biomarker.	CRC stages I–IV
	[37]	To evaluate the feasibility of fecal miRNAs as biomarkers for colorectal neoplasia screening	Stool	RNA extraction from fresh stool specimens and FOBT samples - Direct microRNA analysis (DMA) to assess the feasibility of direct miRNA expression detection from fresh stool specimens - MicroRNA microarray expression profiling and data analysis - MicroRNA quantification by real-time RT-PCR.	Stool miRNA extraction and expression were highly reproducible over time in healthy individuals. In 29 patients, miR-21 and miR-106a levels were elevated in those with CRC or adenomas compared with controls, suggesting these miRNAs could serve as non-invasive biomarkers for colorectal neoplasia detection.	Stage not reported
	[39]	To examine the utility of miRNAs for colon cancer screening in multiethnic populations	Serum	- Vesicle Extraction - RNA extraction from sera - Cdna synthesis - qPCR	When compared to controls, the ratios of miRs-21, miR-29a, and miR-92a’s non-vesicular to extracellular vesicular levels were statistically and quantitatively associated with the stage of CRC.	Stage I–IV
	[40]	To evaluate the level of expression of a miRNA panel in the serum of patients diagnosed with CRC in comparison to the serum of healthy patients, and to find potential and applicable biomarkers	Serum	Total RNA isolation from serum samples - cDNA synthesis - RT-PCR to determine the expression of 179 circulating miRNA isoforms in serum	Several miRNAs were differentially expressed in colorectal tumor tissue. Specifically, miR-324-3p, miR-125b-5p, miR-199a-5p, miR-29c-3p, and miR-30e-5p were significantly downregulated in tumors compared with normal tissue. Most other analyzed miRNAs showed no significant changes. The study highlights these tissue-based miRNAs as potential biomarkers, but serum or exosome levels were not reliably predictive, indicating further validation is needed for non-invasive CRC detection.	Not reported by stage
Proteins						
	[41]	To identify and validate a panel of protein-based biomarkers in independent cohorts that could be translated to a reliable, non-invasive blood-based screening test.	Blood	- ELISA kits to measure biomarkers. - Bead-based assays to analyze IL6 and IL8.	In two independent cohorts (n = 145 and n = 197), seven serum biomarkers showed significant differences between CRC patients and controls, but were individually insufficient for diagnosis. Logistic regression identified a three-biomarker panel—IGFBP2, DKK3, and PKM—that achieved 73% sensitivity and 95% specificity. This panel outperformed fecal occult blood testing in detecting early-stage (Stage I–II) CRC, highlighting its potential as a non-invasive blood-based screening tool.	Stage I–II higher sensitivity
	[42]	To analyze the diagnostic value of a panel of biomarkers, namely Hb, M2-PK, MMP-2, MMP-9, IL-6, and TNF-α for CRC detection in stool samples from patients with CRC, advanced adenoma, and other lesions, as well as healthy patients as controls.	Stool	- FIT-Hb was used to detect occult hemoglobin in stools. - A sandwich ELISA measured M2-PK levels. - Commercial ELISA kits (TNF-α and IL-6) and antibody pairs (MMP-2 and MMP-9) were used to quantify these biomarkers in stool samples.	MMP-2, MMP-9, IL-6, and TNF-α were not useful for CRC diagnosis. Combining FIT-Hb with stool M2-PK improved screening performance: fecal Hb alone showed 92% sensitivity compared to 55% for M2-PK. Considering either fecal Hb ≥ 10 µg/g or M2-PK ≥ 8 U/mL as positive increased sensitivity to 97%, while requiring both markers to be positive increased specificity to 94%.	Advanced adenoma and CRC included
	[43]	To evaluate the use of fecal tumor M2-PK in detection of colorectal cancer in symptomatic adult subjects who underwent colonoscopy	Stool	- A commercially available rapid test, ScheBo M2-PK Quick, was used to test for M2-PK in stool samples	In 85 participants, the stool M2-PK test detected CRC with 100% sensitivity and 72.5% specificity, outperforming gFOBT in sensitivity (64.7%) but with lower specificity (88.2%). Positive and negative predictive values were 47.2% and 100% for M2-PK, compared with 57.9% and 90.9% for gFOBT. The study shows M2-PK is a highly sensitive stool biomarker for CRC detection, though specificity is slightly lower than gFOBT.	Symptomatic adults: stage not specified
Gene Panels/Multi-gene Assays						
	[47]	To examine promoter methylation of two previously identified stool markers (NDRG4 and GATA5 v and two novel markers, namely FOXE1 and SYNE1, as potential biomarkers for the early detection of colorectal cancer in blood DNA	Blood Plasma	- DNA isolation from plasma - Sodium bisulfite treatment - qMSP - Cell culture and transfections for human CRC cell lines - Colony formation assay - In vitro cell proliferation, migration, and invasion assays	Plasma DNA methylation of NDRG4, GATA5, FOXE1, and SYNE1 showed sensitivities of 27%, 18%, 46%, and 47% and specificities of 95%, 99%, 93%, and 96%, respectively, in CRC patients versus controls. Combining FOXE1 and SYNE1 increased sensitivity to 56% with 90% specificity in the training set and 58% sensitivity with 91% specificity in the test set. Functional assays showed FOXE1 overexpression reduced colony formation, while SYNE1 had no significant effect. These results suggest FOXE1 and SYNE1 are potential biomarkers for CRC detection.	All CRC stages
	[46]	To evaluate the performance of proven hypermethylated DNA promoter regions as plasma-based biomarkers for CRC detection	Blood	- DNA extraction from plasma - Sodium bisulfite treatment - PCR	Individual DNA promoter regions had low sensitivity (<30%) for CRC detection. Combining seven hypermethylated promoters (ALX4, BMP3, NPTX2, RARB, SDC2, SEPT9, VIM) with age and sex increased sensitivity to 90.7% and specificity to 72.5%, demonstrating the potential of a multitarget methylation panel for colorectal cancer screening.	Early- & late-stage
	[44]	To demonstrate that screening for CRC or cancer detection in stool specimens collected non-invasively does not require the inclusion of an excessive number of genes, and colonic defects can be identified via the detection of an aberrant protein in the mucosa or submucosa	Stool	- Microarray hybridizations. - RNA purification from stool mud. - RNA quantification. - cDNA synthesis. - RT-PCR to quantify genes of interest. - Immunohistochemistry using COC1021 tissue arrays.	In CRC tissues, UBE2N, IMPDH1, and DYNC1LI1 were upregulated, while HRASLS2 was downregulated. Using a four-gene stool panel, the study achieved 96.6% sensitivity and 89.7% specificity, indicating that this panel can accurately detect colorectal cancer from stool samples.	Early- & late-stage

CRC: colorectal cancer; RNA: ribonucleic acid; miRNA: microRNA; ROC curve: Receiver Operating Characteristic curve; gFOBT: guaiac-based fecal occult blood test.

**Table 3:** A summary highlighting the optimal stool and serum biomarkers identified in the review, along with their reported sensitivity and specificity.

Biomarker/Panel	Sample Type	Sensitivity (%)	Specificity (%)	Reference
DNA Methylation				
Hypermethylated promoter regions (ALX4, NPTX2, BMP3, SDC2, RARB, VIM, SEPT9)	Blood	90.7	72.5	[46]
NDRG4 methylation	Stool	85.7	70.8	[30]
Composite ctDNA methylation (SEPT9 + SDC2 + BCAT1)	Blood	83.7	93.9	[33]
NDRG4 methylation	Stool	76.2	89.1	[32]
NDRG4	Blood	54.8	78.1	[32]
miRNA				
miR-182	Serum	98.0	96.0	[38]
miR-223	Serum	97.1	96.7	[38]
miR-21	Stool	88.1	81.6	[40]
miR-21	Blood	86.05	72.97	[40]
**Protein**				
MMP-9	Stool	72.2	95.0	[42]
MMP-9				
Protein biomarker panel (IGFBP2 + DKK3 + PKM2)	Serum	73.0	95.0	[41]

These tests represent the most promising options according to sensitivity and specificity from the systematic review, providing valuable non-invasive tools for CRC screening.

## Data Availability

The findings presented in this study are available on request from the corresponding author.

## Abbreviations

The following abbreviations are used in this manuscript:

AA	Advanced Adenoma
ADHFE1	Alcohol Dehydrogenase, Iron-Containing 1
ALX4	ALX Homeobox 4
AUC	Area Under the Curve
BCAT1	Branched-Chain Amino Acid Transaminase 1
BMP3	Bone Morphogenetic Protein 3
CRC	Colorectal Cancer
ctDNA	Circulating Tumor DNA
DKK3	Dickkopf-Related Protein 3
DNA	Deoxyribonucleic Acid
DYNC1LI1	Dynein Cytoplasmic 1 Light Intermediate Chain 1
FIT	Fecal Immunochemical Test
FOXE1	Forkhead Box Protein E1
F. nucleatum	Fusobacterium nucleatum
GATA5	GATA Binding Protein 5
gFOBT	Guaiac-based Fecal Occult Blood Test
HRASLS2	Phospholipase A and Acyltransferase 2
IGFBP2	Insulin-like Growth Factor Binding Protein 2
IL-6	Interleukin 6
IMPDH1	Inosine Monophosphate Dehydrogenase 1
LTE-qMSP	Linear Target Enrichment Quantitative Methylation-Specific Polymerase Chain Reaction
MAPK	Mitogen-Activated Protein Kinase
miRNA/miR	MicroRNA
MMPMatrix	Metalloproteinase
mRNA	Messenger Ribonucleic Acid
NDRG4	N-Myc Downstream-Regulated Gene 4
NPTX2	Neuronal Pentraxin 2
PCR	Polymerase Chain Reaction
PKM2	Pyruvate Kinase M2
PPP2R5C	Protein Phosphatase 2 Regulatory Subunit B′γ
qMSP	Quantitative Methylation-Specific Polymerase Chain Reaction
RARB	Retinoic Acid Receptor Beta
RNA	Ribonucleic Acid
SDC2	Syndecan 2
SEPT9/Septin9	Septin 9
SFRP2	Secreted Frizzled-Related Protein 2
SHOX2	Short Stature Homeobox 2
SYNE1	Spectrin Repeat Containing Nuclear Envelope Protein 1
TNF-α	Tumor Necrosis Factor Alpha
UBE2N	Ubiquitin-Conjugating Enzyme E2 N
VIM	Vimentin
WIF-1	Wnt Inhibitory Factor 1
